# Beef and Ice Cream

**Published:** 1879-07

**Authors:** James I. Tucker


### ARTICLE VIII.

*Beef and Ice Cream.*

The following is an article of dietary importance. I believe it originated with me. It is simple, novel and speaks for itself so far as its efficaciousness is concerned. I combine ice-cream and beef so as to make a homogeneous mass. These are about the properties: 120 grams cream, 30 grams sugar, 8 grams ext. vanilla, 8 grams beef juice ("Johnston's" I have generally used, but the juice squeezed from beef steak is just as good). Any confectioner can make it extemporaneously (or within an hour) or it can be made at home at short notice. It has done me excellent service.

JAMES I. TUCKER, M.D.

---

THE WOMAN'S MEDICAL COLLEGE.—Dr. Wm. J. Maynard has been elected Professor of Materia Medica, Therapeutics and Dermatology; Dr. Wm. T. Montgomery has been elected Professor of Ophthalmology and Otology, and Dr. E. Fletcher Ingals has been elected Professor of Diseases of the Chest and Throat in this institution. These are wise selections; they will add to the strength and prosperity of the College.

R. S. DEWEY, M.D., 1st Assistant Physician of the Hospital for the Insane at Elgin, has been appointed to the superintendency of the new Hospital for the Insane at Kankakee—a good selection.

